# Efficacy of topical application of zinc compounds for oral mucosal diseases: A systematic review and meta-analysis

**DOI:** 10.4317/medoral.27549

**Published:** 2025-11-22

**Authors:** Moon-Jong Kim, Hong-Seop Kho

**Affiliations:** 1 Department of Oral Medicine, Gwanak Seoul National University Dental Hospital, 1 Gwanak-ro, Gwanak-gu, Seoul 08826, South Korea (ROK); 2 Department of Oral Medicine and Oral Diagnosis, School of Dentistry and Dental Research Institute, Seoul National University, 101 Daehak-ro, Jongno-gu, Seoul 03080, South Korea (ROK); 3 Institute of Aging, Seoul National University, Seoul, South Korea (ROK)

## Abstract

**Background:**

Zinc has been used as a topical therapeutic agent for the management of various oral and dermatological conditions. The purpose of this systematic review was to evaluate the effectiveness of topical zinc therapy in the prevention and treatment of oral mucosal diseases.

**Material and Methods:**

A comprehensive literature search across four databases identified studies evaluating the effects of topical zinc therapy on oral mucosal diseases. Two independent reviewers evaluated the identified studies, extracting relevant data. Risk of bias assessment and meta-analysis were performed for randomized controlled trials (RCTs).

**Results:**

This systematic review included 14 studies: eight on oral mucositis, three on recurrent aphthous stomatitis (RAS), two on oral lichen planus (OLP), and one on herpes simplex virus (HSV) infection. Among them, seven studies on oral mucositis, two on RAS, and two on OLP were RCTs. The oral mucositis studies demonstrated that topical zinc therapy significantly alleviated cancer therapy-induced oral mucositis, with meta-analysis confirming significant improvement at weeks 2, 3, and 4 compared with control treatments. For RAS, topical zinc therapy reduced pain intensity and ulcer size although significant differences between treatment and control groups were observed in only one RCT. For OLP, two RCTs reported symptom improvement although treatment outcomes varied. Additionally, a case series has suggested the potential benefit of topical zinc in mitigating HSV recurrence.

**Conclusions:**

Topical zinc therapy has promising efficacy in managing oral mucosal diseases, particularly in cancer therapy-induced oral mucositis. However, evidence for other conditions remains limited. Further comprehensive RCTs are needed to establish its effectiveness across various oral mucosal diseases.

## Introduction

Oral mucosal diseases encompass various pathological conditions affecting the oral cavity's mucosal tissues, caused by local and/or systemic factors. These conditions often cause significant pain and discomfort, impairing essential daily functions such as eating and speaking, thereby reducing quality of life (1). They are particularly prevalent among older adults due to contributing factors such as systemic diseases, polypharmacy, xerostomia, and denture use (2 - 6). With the aging population increasing, effective management strategies for oral mucosal diseases are becoming increasingly important.

The primary treatment approach for oral mucosal diseases including oral lichen planus (OLP), recurrent aphthous stomatitis (RAS), oral mucositis, and oral candidiasis remains pharmacological, primarily involving corticosteroids and antimicrobial agents. However, due to the chronic and recurrent nature of these conditions, long-term medication use is common, raising concerns about adverse drug reactions and drug resistance issues, particularly in older adult patients (7). Additionally, many older adult patients experience polypharmacy, increasing the risks of drug interactions and potential contraindications (6). For instance, corticosteroids can induce secondary oral candidiasis as an adverse effect. Azole antifungal agents used for oral candidiasis have well-known drug interactions through the inhibition of cytochrome P450 enzymes and are contraindicated in patients with hepatic or renal impairment. Therefore, there is a growing need for alternative therapies with improved safety profiles and sustained efficacy.

Zinc is an essential trace element involved in immune regulation, oxidative stress responses, and tissue repair (8 - 10). It also has antimicrobial properties, including antibacterial, antifungal, and antiviral effects (11). Zinc deficiency may impair these functions, leading to delayed wound healing, reduced immune defenses, and increased risk of systemic diseases such as diabetes, cardiovascular diseases, and chronic inflammatory bowel disease (12 - 14). In the oral cavity, zinc deficiency is linked to taste disorders and greater susceptibility to periodontal and oral mucosal diseases (15 , 16). Animal studies further suggest that zinc deficiency contributes to periodontal breakdown and impaired mucosal integrity (17), and clinical studies have frequently reported a zinc deficiency in patients with RAS (18).

Given its critical roles in physiological and pathological processes, several attempts have been made to utilize zinc as a therapeutic agent. Zinc compounds are widely used to treat various dermatological conditions (9) and have been explored as adjunct therapies for neurological disorders and cancer treatment (10 , 19). In oral health, zinc has been studied for its role in promoting periodontal health and preventing dental caries (17 , 20). Additionally, zinc supplementation has been used to alleviate symptoms in patients with zinc deficiency-related taste disorders and oral mucosal discomfort (21 , 22). Furthermore, its therapeutic benefits in RAS management support its potential application in treating oral mucosal diseases (16 , 23).

Topical zinc applications are considered safe and generally better tolerated than systemic use (24). In oral health, topical zinc is particularly effective, as it provides direct therapeutic effects while minimizing systemic absorption. Numerous studies have reported its efficacy in treating oral mucosal diseases (25 - 38). Despite these promising findings, further research is required to establish the efficacy of topical zinc therapy for oral mucosal diseases, as existing studies vary in design, zinc formulations, and outcome measures. A systematic review and meta-analysis are therefore necessary to evaluate its therapeutic potential. While previous reviews have examined the effects of systemic zinc supplementation on oral mucosal diseases (39 , 40), no comprehensive systematic review and meta-analysis has specifically evaluated the therapeutic benefits of topical zinc applications. Therefore, this study aimed to systematically evaluate its clinical efficacy in the treatment and prevention of oral mucosal diseases.

## Material and Methods

This systematic review was conducted in accordance with the Preferred Reporting Items for Systematic Reviews and Meta-Analyses (PRISMA) guidelines (41). The review protocol has been registered with the International Prospective Register of Systematic Reviews (PROSPERO) (registration number: CRD42024622783). The PICO question for this systematic review was defined as follows: Population (P) -patients with oral mucosal diseases; Intervention (I) -zinc used as a topical agent; Comparison (C) -no treatment or other treatments (or absence of a control group, if applicable); Outcome (O) -treatment effects such as lesion size reduction, pain reduction, and other relevant clinical outcomes.

Literature search strategy

A comprehensive literature search was performed across four online databases-PubMed, Embase, Cochrane Library, and Scopus-to identify relevant studies on the efficacy of topical zinc compounds for oral mucosal diseases. The search included studies published up to December 2024 across all databases, with no restrictions on the starting date. The following keywords and search terms were used: Intervention-related terms: Zinc OR topical OR mouthwash OR mouth rinse OR ointment OR spray OR toothpaste OR cream; Disease-related terms: Oral mucosal diseases OR aphthous stomatitis OR oral ulcers OR stomatitis OR oral lichen planus OR oral candidiasis OR burning mouth syndrome OR xerostomia OR dry mouth.

Eligibility criteria

Studies were included if they met the following inclusion criteria: Investigated the effects of topical zinc compounds on oral mucosal diseases; published in English; conducted on human subjects and published in peer-reviewed scientific journals. All study designs-including case reports, and randomized controlled trials (RCTs)-were considered for inclusion.

Studies were excluded if they met any of the following exclusion criteria: Not conducted on human subjects; did not target oral mucosal diseases (e.g., studies focusing on periodontitis, dental caries, or halitosis); review articles; published in languages other than English and full-texts not accessible.

Screening and selection procedures

The initial search results were imported into EndNote, a reference management software, to organize citations and remove duplicates. Two independent reviewers (MJK and HSK) screened the titles and abstracts to assess eligibility based on predefined inclusion and exclusion criteria. Studies that appeared to meet the inclusion criteria were subsequently retrieved for full-text review. During this phase, any disagreements between the two reviewers were resolved through discussion until consensus was reached.

Data extraction

Data extraction was performed to collect relevant information from the included studies. One reviewer (MJK) extracted the data, and another reviewer (HSK) independently reviewed it for accuracy. Any discrepancies were resolved through discussion. The extracted data included the following details: First author, year of publication, country where the study was conducted, study design, targeted oral mucosal diseases, type of zinc compound used (formulation and concentration), use of concomitant medications, study duration, number of participants and their mean age, use of control group medication and its type, and the criteria and outcomes for treatment efficacy.

Risk of bias assessment

The risk of bias in the included RCTs was assessed using the Cochrane Risk of Bias Tool 2 (RoB 2.0) (42). This tool evaluates five domains, including bias from the randomization process, bias due to deviations from intended interventions, bias due to missing outcome data, bias in outcome measurement, and bias in the selection of reported results. The risk of bias assessment was performed by one reviewer (MJK) and independently reviewed by another reviewer (HSK). Any discrepancies were resolved through discussion.

No formal risk of bias assessment was performed for non-randomized studies due to their limited number and methodological heterogeneity.

Data analysis

Data analysis was conducted using Review Manager (RevMan) version 5.4. Only RCTs were included in the meta-analysis. No data conversion procedures were applied prior to analysis. When data were presented only in graphical form, numerical estimates were extracted. Studies were excluded from meta-analysis when necessary outcome data could not be obtained or when fewer than two studies were available for a given condition. For continuous variables, either the Standardized Mean Difference (SMD) or Mean Difference (MD) was applied, depending on the consistency of the measurement scales across studies. To evaluate heterogeneity among the included studies, I² statistics and Cochran's Q test were used. An I²&gt;50% indicated substantial heterogeneity. A fixed-effects model was applied when statistical heterogeneity was low (I²&lt;50%). In contrast, a random-effects model was applied when statistical heterogeneity was high (I²50%).

## Results

Literature search

**Figure 1:**
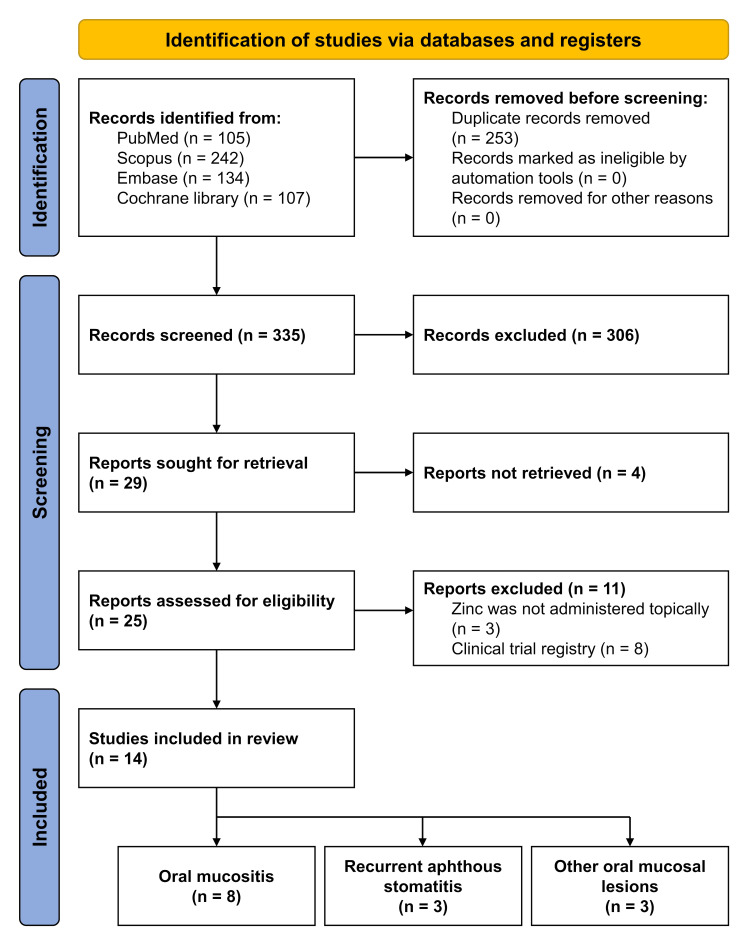
PRISMA flow diagram of the study selection process.

A total of 588 studies were identified from the database searches, including PubMed (n=105), Scopus (n=242), Embase (n=134), and Cochrane Library (n=107). After removing 253 duplicate articles, 335 records were retained for screening. The titles and abstracts of these articles were screened based on the predefined inclusion and exclusion criteria, leading to the exclusion of 306 articles. Among the 29 studies sought for full-text retrieval, 4 were not retrieved. The remaining 25 studies underwent full-text review, with 11 excluded because they did not administer zinc topically or were clinical trial registries. Ultimately, 14 studies were included in the systematic review. The PRISMA Flow Diagram (Figure 1) illustrates the study selection process and the exclusion criteria.

1

Study characteristics

Between 1981 and 2024, a total of 14 studies were included in this review, investigating the effects of topical zinc therapy on oral mucositis, RAS, OLP, and herpes simplex virus (HSV) infection.

The eight studies on oral mucositis investigated the therapeutic effects of topical zinc therapy in patients undergoing radiotherapy or chemotherapy. Six studies enrolled patients who had not yet developed oral mucositis at the start of treatment (28 , 30 , 32 - 34 , 37), whereas the others enrolled patients who had already developed oral mucositis during treatment (36 , 38). Most of these studies were RCTs, with only one observational study (32). Sample size was 30-145 participants, with experimental (aged 46-65 years) and control (aged 46-68 years) groups. However, three RCTs did not report the mean participant age (28 , 36 , 38). The studies evaluated various zinc compounds, including zinc sulfate (28 , 37 , 38), zinc oxide (30), zinc chloride (33 , 34), polyvinylpyrrolidone-zinc gluconate (32), and zinc carnosine (36). These compounds were delivered as mouthwashes (28 , 33 , 34 , 37 , 38), paste (30), spray (32), or gel (36) at concentrations of 0.2%-5%. Control interventions included chlorhexidine, sodium bicarbonate, polyherbal solutions, benzydamine, and placebo. Follow-up periods varied across studies, with a maximum duration of eight weeks. The primary outcome in most studies was oral mucositis severity, assessed using the WHO grading system, the Spijkervet scale (28), and the Oral Mucositis Assessment Scale (37). The other outcomes assessed included quality of life, pain intensity, body weight, oral mucositis prevention, and healing duration (Table 1 and Table 2).

Table 1 Table 2

The remaining six studies included two RCTs (29 , 31) and one pilot clinical study (26) on RAS, two RCTs (27 , 35) on OLP, and one case series (25) on HSV infection. Sample size was 5-64 participants. Only one study (31) reported the mean participant age, with an average of 38 years in the experimental group and 41 years in the control group. The studies evaluated different zinc compounds, including zinc sulfate (25 , 26 , 31), zinc oxide (35), and unspecified zinc compounds (27 , 29). These were delivered as mouthwashes (25 - 27 , 29), nanofibrous mats (35), or mucoadhesive tablets (31). In the RCTs, the control groups received either triamcinolone ointment or placebo treatments. In some RCTs on RAS and OLP, topical steroid ointments were used concurrently as a standard treatment in both the experimental zinc and control groups (27 , 29). The study durations ranged from 1 week to 23 months (Table 3 and Table 4).

Table 3 Table 4

Across the included RCTs, the dropout rates were 6.1% (34/557) in studies on oral mucositis, 5.7% (4/70) in studies on RAS, and 0% (0/40) in studies on OLP, resulting in 523, 66 and 40 patients being included in the final analyses..

Quality assessment

**Figure 2:**
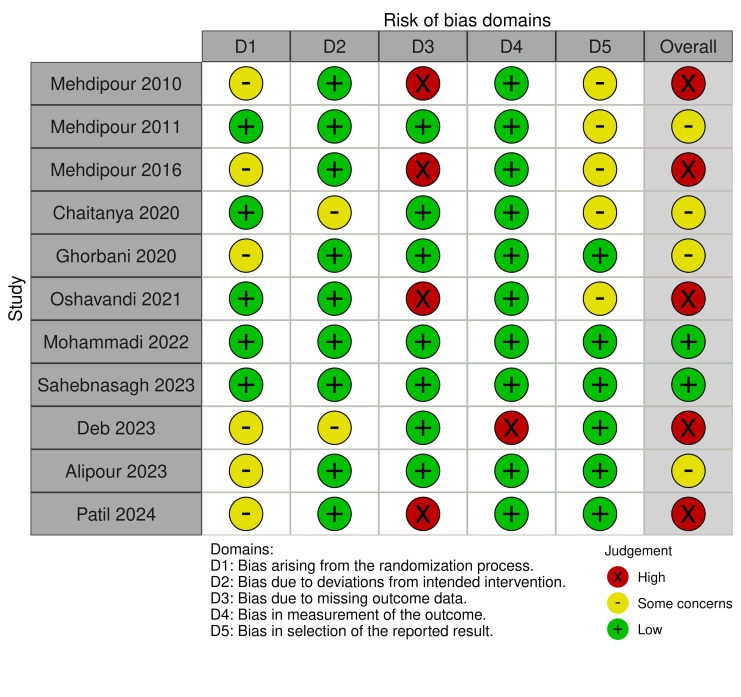
Risk of bias assessment across the five domains for the included randomizedcontrolled trials using Cochrane RoB 2.0.

**Figure 3:**
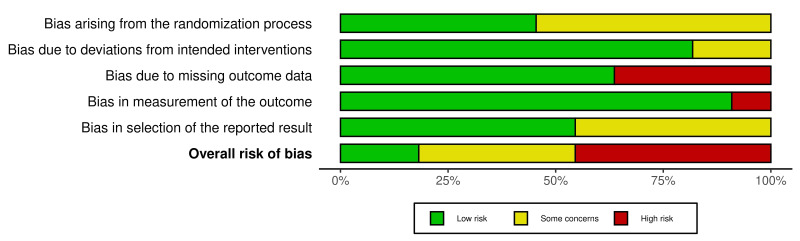
Summary of risk of bias judgments across the five domains for the included randomized controlledtrials using the Cochrane RoB 2.0.

Most studies had at least some concern or high risk of bias in one or more domains (Figure 2 and Figure 3). The most notable issue was bias due to missing outcome data (D3), with four studies rated as high risk, either due to a high dropout rate (33) or insufficient details about missing outcome data (27 , 29 , 38). Additional concerns were identified by six studies (27 , 29 , 31 , 35 , 36 , 38) regarding the randomization process (D1) and by five studies (27 - 30 , 33) regarding compliance with pre-specified analysis plans (D5) due to missing information on whether a pre-specified analysis plan was established and followed. In contrast, bias due to deviations from intended interventions (D2) and bias due to outcome measurement (D4) were generally assessed as low risk. However, Chaitanya et al. (30) rated D2 as a concern due to missing information on allocation concealment. Additionally, an open-label study by Deb et al. (36) was rated a concern for D2 and a high risk for D4.

2

3

Considering the overall risk of bias, most studies were categorized as some concern or high risk, with only two studies (34 , 37) rated as low risk. Consequently, the results should be carefully interpreted, as bias across multiple studies may influence the estimated effects of topical zinc therapy for oral mucosal diseases.

Main findings

Oral mucositis

Eight studies-seven RCTs and one observational study-investigated the therapeutic effects of topical zinc therapy on oral mucositis in patients undergoing chemotherapy or radiotherapy for various cancers. The observational study demonstrated that topical zinc spray effectively alleviated oral mucositis in a substantial proportion of patients. Across the RCTs, patients treated with topical zinc consistently exhibited significantly lower oral mucositis severity compared with the control groups at most assessment points.

**Figure 4:**
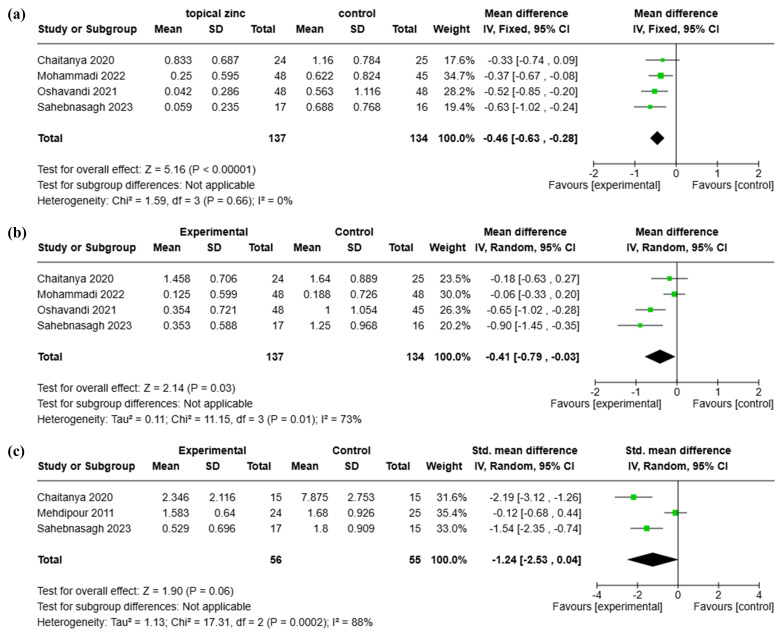
Forest plots of meta-analyses assessing the effects of topical zinc therapy on the severity of oralmucositis at (a) 2, (b) 3 and (c) 4 weeks post-treatment initiation.

A meta-analysis of five RCTs evaluated the effect of topical zinc therapy on oral mucositis severity. Two RCTs were excluded: Deb et al. (36) did not assess oral mucositis severity, whereas Patil et al. (38) reported results using medians instead of means and lacked clear data on treatment duration. The analysis focused on three time points (2, 3 and 4 weeks), as these were the most commonly assessed intervals. At 2 weeks, topical zinc therapy significantly reduced oral mucositis severity compared with control (MD= -0.46, 95% CI: -0.63 - -0.28; p&lt;0.00001; I²=0%) (Figure 4a). At 3 weeks, the benefit remained significant (MD= -0.41, 95% CI: -0.79 - -0.03; p=0.03; I²=73%) (Figure 4b). At 4 weeks, although zinc continued to show favorable effects, statistical significance was marginal (SMD= -1.24, 95% CI: -2.53 -0.04; p=0.06; I²=88%) (Figure 4c). A sensitivity analysis excluding the high-risk study (33) did not substantially alter the results of the meta-analysis (data not shown). Due to differences in evaluation metrics and analytical methods across the time points, the trends in treatment efficacy over time were inaccessible.

4

Recurrent aphthous stomatitis

Three studies-one pilot clinical study and two RCTs-investigated the efficacy of topical zinc therapy for RAS management. The pilot clinical study (26) reported symptom improvement in approximately 70% of patients after using a zinc gargle for two months. The two RCTs, Mehdipour et al. (29) and Ghorbani et al. (31), both observed reductions in pain intensity and ulcer size after using a zinc gargle for two weeks and a zinc mucoadhesive tablet for one week, respectively. However, Mehdipour et al. (29) found no significant differences between the experimental and control groups, possibly due to concurrent triamcinolone ointment use in both groups, which may have confounded the effects of zinc therapy.

Despite the inclusion of RCTs, a meta-analysis was not performed due to the limited number of RCTs (n=2) and incomplete outcome data reported by Mehdipour et al. (29).

Other oral mucosal diseases

Two RCT investigated the effects of topical zinc therapy on OLP using different zinc formulations (27 , 35). Both studies reported reductions in lesion size and pain intensity, but the significance of these improvements varied between studies. Mehdipour et al. (27) reported that lesion size reduced significantly in the zinc group compared with the control group, whereas pain intensity decreased similarly in both groups. Alipour et al. (35) reported that pain intensity was significantly lower in the zinc group than in the control group, whereas lesion size reduction was indifferent between the groups. These differences are likely attributable to the variations in control treatments (triamcinolone ointment versus placebo) used between studies. Additionally, the therapeutic effects of topical zinc in OLP may depend on its formulation and delivery method, influencing whether lesion healing or symptom relief becomes the primary treatment outcome. As with RAS, a meta-analysis for OLP was not performed due to the limited number of RCTs (n=2) and incomplete outcome data in Mehdipour et al. (27).

An observational study investigated the preventive effects of a low-concentration zinc sulfate solution in 25 patients with recurrent HSV infection, including five with oral mucosal lesions (25). The application of the zinc solution successfully prevented HSV relapses in the oral mucosa over a 16-23-month observation period. This study highlights the potential prophylactic benefits of topical zinc therapy for managing herpes simplex-related oral mucosal lesions.

## Discussion

Oral mucosal diseases significantly impact patients' quality of life by causing pain, discomfort, and functional impairment. While pharmacological treatments, including corticosteroids and antimicrobials, are commonly used, their long-term use may lead to adverse effects, particularly in elderly or medically compromised patients. Therefore, safer and more targeted adjunctive therapies are needed. This systematic review evaluated the therapeutic potential of topical zinc therapy for various oral mucosal diseases and highlighted three main findings. First, topical zinc therapy demonstrated consistent benefits in reducing the severity of cancer therapy-induced oral mucositis. Second, for RAS and OLP, zinc therapy was generally associated with symptom improvement, although the evidence remained limited. Third, evidence for other oral mucosal diseases such as HSV infection was scarce, but a preliminary report suggested potential benefit. These findings together supported the potential of topical zinc therapy as a complementary treatment modality for oral mucosal diseases.

Oral mucositis is a common complication of cancer therapy, affecting nearly all patients undergoing chemotherapy or radiotherapy for head and neck cancers (43). Clinically, it presents as widespread erythema and ulceration of the oral mucosa, accompanied by severe pain that interferes with oral intake and significantly diminishes quality of life (44). In severe cases, bleeding and an increased risk of secondary infections may occur, necessitating treatment modifications, compromising cancer therapy effectiveness. These complications lead to prolonged hospitalization and higher healthcare costs, significantly burdening both patients and healthcare systems economically (45). Therefore, effective prevention and management strategies for oral mucositis are essential. Although recent guidelines recommend various interventions, including basic oral hygiene care, anti-inflammatory agents, photobiomodulation therapy, and cryotherapy, no universally established treatment exists despite extensive research efforts (46).

To our knowledge, this is the first meta-analysis to quantitatively assess the efficacy of topical zinc therapy against cancer therapy-induced oral mucositis. The results demonstrated a consistent trend favoring zinc over control interventions in reducing mucositis severity at 2, 3, and 4 weeks post-treatment initiation. Statistically significant benefits were observed at 2 and 3 weeks, and the largest effect size was observed at 4 weeks, although the result narrowly missed statistical significance, possibly due to the limited sample size and high heterogeneity rather than a lack of therapeutic benefit. These findings suggest that topical zinc therapy provides clinical benefits during cancer treatment, although the evidence is limited by heterogeneity and potential risk of bias.

The development of oral mucositis during cancer therapy follows a complex, multistep process involving direct epithelial injury, inflammatory signaling, delayed tissue repair, and eventual healing, as outlined in a five-phase model (47). Recent studies have further emphasized the roles of proinflammatory cytokines, oxidative stress, tight junction disruption, and microbiome alterations in aggravating mucosal damage (48). Zinc may exert therapeutic effects by regulating inflammatory cytokine production and promoting antioxidant activity, thereby interfering with key mechanisms involved in the onset and progression of oral mucositis (8 , 10). Additionally, zinc protects the epithelial barrier, potentially preventing epithelial cell apoptosis and detachment (49). These mechanisms may explain the clinical benefits observed in patients receiving topical zinc therapy for cancer therapy-induced oral mucositis.

Besides oral mucositis, this review examined studies investigating topical zinc therapy for RAS and OLP. However, a meta-analysis was not feasible due to the small number of studies and variations in study design, zinc formulation, and treatment duration. Nonetheless, topical zinc therapy was generally associated with reduced lesion size and decreased pain intensity. Both RAS and OLP are recurrent or chronic inflammatory conditions driven by dysregulated cell-mediated immune responses (50 - 52), and zinc may provide therapeutic benefits in these conditions. However, for OLP, the two included studies reported conflicting results regarding statistical significance across outcome measures. This discrepancy is likely due to differences in zinc formulations and the use of concomitant medications.

Reportedly, zinc has antibacterial, antifungal, and antiviral properties (11). However, clinical evidence supporting topical zinc therapy for infectious oral mucosal diseases, such as oral candidiasis and HSV infection, remains limited. This review identified only one case series that reported the preventive effect of topical zinc against HSV infection recurrence (25), highlighting the scarcity of research in this area. Considering zinc's favorable safety profile and potential antifungal activity (53 - 55), further studies are warranted to explore its role in managing oral candidiasis, particularly in aging populations, where such conditions are increasingly prevalent.

This review has several limitations that should be considered when interpreting the findings. First, the small number of included studies precluded subgroup analyses based on zinc type and concentration. Consequently, it remains unclear which specific formulations or dosing strategies are most effective. Additionally, most of the included studies focused on cancer therapy-induced oral mucositis, emphasizing the need for broader research on other oral mucosal diseases. Second, the overall methodological quality of the included studies was suboptimal. Many of the included RCTs were rated as having some concerns or high risk of bias according to the RoB 2.0 assessment, warranting caution in interpreting the results. Third, most of the included studies were conducted in Iran and India, leading to a notable geographic concentration that may affect the generalizability of the findings, as differences in genetic background, environmental exposures, and healthcare systems could influence treatment response. Fourth, this review included only studies published in English and did not incorporate gray literature, which may have introduced selection or publication bias. Furthermore, although our findings suggest that topical zinc therapy may be beneficial for managing oral mucositis, there is currently insufficient evidence to determine whether zinc therapy is superior to standard treatments such as corticosteroids. Further well-designed comparative studies with larger sample sizes are needed to clarify the relative effectiveness of zinc in the management of oral mucositis.

## Conclusions

This systematic review and meta-analysis suggests that topical zinc therapy has therapeutic potential in managing oral mucosal diseases, particularly in reducing the severity of cancer therapy-induced oral mucositis. While evidence for its application in RAS, OLP, and HSV infection remains limited, the observed clinical improvements and favorable safety profile highlight zinc's potential as an adjunctive treatment option. Further well-designed studies are needed to confirm these findings and establish optimal formulations and dosing for topical zinc therapy across various oral mucosal diseases.

## Figures and Tables

**Table 1:** Table Characteristics of included studies on oral mucositis.

Study	Country	Study design	Sample size*	Study duration
Mehdipour (2011)	Iran	RCT	30 (15/15)	8 weeks
Chaitanya (2020)	India	RCT	75 (25/25/25)	~35 days
Niculita (2021)	Romania	Observational study	145a	24 days
Oshavandi (2021)	Iran	RCT	96 (48/48)	3 weeks
Mohammadi (2022)	Iran	RCT	144 (48/48/48)	3 weeks
Sahebnasagh (2023)	Iran	RCT	67 (17/17/17/16)	6 weeks
Deb (2023)	India	RCT	45 (15/15/15)	Until healed (up to 10 days)
Patil (2024)	India	RCT	100 (50/50)	15 days

* Sample size is presented as Total (Experimental group/Control group 1/Control group 2...)a No control group. RCT: Randomized controlled trial.

**Table 2:** Table Intervention and outcomes of included studies on oral mucositis.

Study	Zinc compound	Control intervention	Outcomes
Mehdipour (2011)	ZnSO4 mouthwash, 0.2%	Chlorhexidine mouthwash	Spijkervet scale
Chaitanya (2020)	ZnO 5% paste Improvised zinc 1%	Standard treatment at cancer hospital	WHO grading system
Niculita (2021)	Polyvinylpyrrolidone-Zn gluconate spray	N/A(no comparative group)	Prevention of OM WHO grading system
Oshavandi (2021)	ZnCl2 mouthwash, 0.2%	Placebo mouthwash	WHO grading systemWeight
Mohammadi (2022)	ZnCl2 mouthwash, 0.2%	NaHCO3 mouthwash, 5%Placebo	WHO grading systemEORTCQLQ-C30
Sahebnasagh (2023)	ZnSO4 mouthwash, 0.2%	PolyherbalChlorhexidinePlacebo	WHO grading systemOMASVAS
Deb (2023)	Zinc carnosine gel, 2%	ChlorhexidineZytee-L	Day of healingVAS
Patil (2024)	ZnSO4 mouthwash, 2.5%	Benzydamine	WHO grading system

EORTCQLQ-C30: European Organization for Research and Treatment of Cancer Quality of Life Questionnaire, OM: Oral mucositis, OMAS: Oral mucositis assessment scale, VAS: Visual analogue scale.

**Table 3:** Table Characteristics of included studies on other oral mucosal diseases.

Study	Country	Study design	Target disease	Sample size*	Study duration
Brody (1981)	Sweden	Case series	HSV infection	5a	16-23 months
Hoogendoorn (1987)	Netherlands	Pilot clinical study	RAS	64a	2 months
Mehdipour (2010)	Iran	RCT	OLP	20 (10/10)	2 months
Mehdipour (2016)	Iran	RCT	RAS	20 (10/10)	2 weeks
Ghorbani (2020)	Iran	RCT	RAS	50 (24/26)	7 days
Alipour (2023)	Iran	RCT	OLP	10 (10/10)b	2 weeks

* Sample size is presented as Total (Experimental group/Control group).a No control group. b Each participant received both the experimental and control treatments on opposite sides of the buccal mucosa.HSV: Herpes simplex virus, OLP: Oral lichen planus, RAS: Recurrent aphthous stomatitis, RCT: Randomized controlled trial.

**Table 4:** Table Intervention and outcomes of included studies on other oral mucosal diseases.

Study	Zinc compound	Control intervention	Outcomes
Brody (1981)	ZnSO4 solution,0.01~0.025%	N/A	Prevention of relapse
Hoogendoorn (1987)	ZnSO4 mouthwash,0.0002%	N/A	Frequency of attack
Mehdipour (2010)	Zinc-containing mouthwash, 0.2%	Placebo mouthwash	VASSize of lesions
Mehdipour (2016)	Zinc-containing mouthwash	Placebo mouthwash	VASNumber, duration and size of ulcer Ulcer-free period
Ghorbani (2020)	ZnSO4-containing mucoadhesive tablet, 5mg	Placebo mucoadhesive tablet	VASSize of ulcer
Alipour (2023)	ZnO-containing nanofibrous mat	Triamcinolone ointment	VASSize of erosion

VAS: Visual analogue scale.

## Data Availability

The datasets used and/or analyzed during the current study are available from the corresponding author.
